# Biological Control of *Fusarium verticillioides* P03 in Maize by *Bacillus cereus sensu lato* B25 Involves Coordinated Host–Bacterium Responses

**DOI:** 10.3390/microorganisms14071517

**Published:** 2026-07-11

**Authors:** Jesús Eduardo Cazares-Álvarez, Karem María Figueroa-Brambila, Alejandro Miguel Figueroa-López, Francisco Roberto Quiroz-Figueroa, Ignacio Eduardo Maldonado-Mendoza

**Affiliations:** 1 SECIHTI—Instituto Politécnico Nacional, Centro Interdisciplinario de Investigación para el Desarrollo Integral Regional (CIIDIR) Unidad Sinaloa, Guasave 81101, SIN, Mexico; eduardo.cazaresalvarez@gmail.com; 2 Departamento de Biotecnología Agrícola, CIIDIR Unidad Sinaloa, Instituto Politécnico Nacional, Guasave 81101, SIN, Mexico; karemfib0318@gmail.com (K.M.F.-B.); fquiroz@ipn.mx (F.R.Q.-F.); 3 Departamento de Ciencias Naturales y Exactas, Universidad Autónoma de Occidente, Unidad Regional Los Mochis, Los Mochis 81217, SIN, Mexico; alejandro.figueroa@uadeo.mx

**Keywords:** chitinase, expression, maize, fungus, bacteria

## Abstract

Maize is a major global crop; however, its production is affected by *Fusarium verticillioides*, which causes stalk, ear, and root rot. *Bacillus cereus* B25 is a maize bacterium that antagonizes *F. verticillioides*, likely through antifungal compounds and possibly by inducing maize chitinase genes. *Fusarium verticillioides* effectively infects maize by producing a chitinase-modifying protein that disrupts maize chitinases, preventing fungal cell wall degradation and evasion of plant immune responses triggered by Pathogen-Associated Molecular Patterns. The aim of this work was to analyze maize, B25, and *F. verticillioides* gene expression during bipartite and tripartite interactions at early stages (5, 7, 10, and 14 days post-inoculation). Physiological results showed increased root and shoot growth in maize seedlings under the tripartite interactions compared to fungus alone. B25 was demonstrated to grow endophytically and coexist with *F. verticillioides* in maize roots. Maize extracellular chitinase genes were induced, possibly due to chitin fragments released from the fungal cell wall, while the fungus effector genes were also upregulated in response. Furthermore, the chitinase gene *Zm00001eb317090* (*bk4*) may contribute to cell wall strengthening, as suggested by in silico co-expression analyses. Overall, these results support a coordinated interaction between maize and B25 that contributes to controlling *F. verticillioides* infection.

## 1. Introduction

*Fusarium verticillioides* (*Fv*) is one of the main pathogenic fungi that affect maize crops. This fungus belongs to the Nectriaceae family, and it belongs to the *Fusarium fujikuroi* species complex (FFSC) [1]. *Fv* is a facultative endophytic fungus which can be found inside the maize plant without presenting any symptoms of infection [2]. In addition, *Fv* can switch to a necrotrophic stage where the fungus biomass increases, resulting in necrosis of infected tissues, and *Fv* infection in grain produces high amounts of mycotoxins (FB1, FB2, and FB3) [3]. FB1 mycotoxin is the most abundant toxin of *Fv* affecting both animal and human health [4]. FB1 inhibits ceramide synthesis and sphingolipid metabolism, causing the accumulation of reactive oxygen species (ROS) and subsequently cell death [5].

In the systemic infection stage of *Fv*, conidia are produced, which can accumulate in the cells of the root vascular tissue and reach the different plant tissues through the stem, causing infection of the entire plant [6]. Since *Fv* is a fungus that can live inside the maize tissues and conidia can form inside the vascular vessels [7], its growth control becomes challenging. Chemical applications of fungicide only limit its infection progress, but do not prevent the fungus from invading the plant or producing mycotoxins. However, biological control has been implemented as an alternative to the use of chemical compounds, which can accumulate in soils and have indirect or direct effects on the environment [8]. Biological control acts using beneficial living microorganisms to prevent or control any fungal infection and this alternative also minimizes pollution by chemical compounds [9]. *Bacillus cereus sensu lato* B25 was isolated from the maize rhizosphere; it was previously shown that B25 is a plant growth-promoting bacterium and controls the *Fv* infection in greenhouse and field conditions [10,11]. B25 has several antagonistic mechanisms against *Fv*, which include genes for production of biofilm, siderophores, antibiotics and lytic enzymes [12]. Both B25 chitinase genes (A and B) are induced by chitin or *Fv* lysates [13], or when B25 is confronted directly with *Fv* [14]; also, both B25 chitinase recombinant proteins inhibit *Fv* conidia germination when applied directly [15].

Maize plant chitinases play an important role as a surveillance mechanism for the presence of fungi and can monitor *Fv* infection; when a fungus is attacking a plant, chitinases degrade chitin on their cell walls, producing chito-oligomers which act as elicitors [16]. These elicitors are available for detection by receptor proteins in plant cells, also called pattern recognition receptors (PRRs) [17]. Among these PRRs, Chitin Elicitor-Binding Protein (CEBiP) plays a crucial role in the perception of chitin-derived elicitors. CEBiP contains extracellular LysM domains that bind chitin oligosaccharides, initiating pattern-triggered immunity (PTI) [18]. This signaling pathway induces defense responses, including reactive oxygen species production and the expression of defense-related genes, thereby enhancing resistance against fungal invasion.

It has been discovered that some fungi can avoid the hydrolysis of their cell wall by plant chitinases by inducing effectors that can detect and modify chitinases, such as fungalysin (*Fv*cmp) from *Fv* [19]. Effectors recognize specific domains of certain extracellular chitinases (*Zm*ChitA and *Zm*ChitB), causing the separation of the chitin-binding domain from the catalytic domain and making maize chitinases unable to bind chitin in the fungal cell wall, and therefore avoiding the release of chitin elicitors (chito-oligosaccharides—COSs), which work as Pathogen-Associated Molecular Patterns (PAMPs) that help the plant to induce its own defense system, which allows *Fv* infection to proceed. On the other hand, several fungal effectors capable of suppressing programmed cell death and the plant hypersensitive response have been reported, including CFEM (Common in Fungal Extracellular Membrane) domain-containing proteins, such as CfEC12 and CgCFEM from *Colletotrichum graminicola* [20,21]. These effectors can also suppress chitin-triggered reactive oxygen species (ROS) accumulation and alter the expression patterns of defense-related genes, such as CgCFEM1 from *Colletotrichum gloeosporioides* [22]. However, despite these advances, the biological functions of CFEM proteins in *F. verticillioides* remain poorly understood.

In a recent study [23], it was demonstrated that ten chitinase genes are induced in 7-day-old maize seedlings when infected with *Fv*, including four genes encoding extracellular chitinases which could be targeted by the fungal fungalysin *Fvc*mp. Nevertheless, although valuable information was obtained recently from transcriptomics data [24], this corresponds to a single time point (7 days post-inoculation); thus, the gene expression responses are still not known during the early developmental stages of a maize plant (first two weeks after inoculation), when bipartite interactions with the fungal pathogen *Fv* or the bacterial control agent of this pathogen, B25, or a tripartite interaction between the plant, *Fv* and B25 takes place.

The present study aims to elucidate the role of the bacterial control agent B25, during a time-course analysis of the first two weeks of bi- and tripartite interactions between maize roots, B25, and *Fv*, in the gene expression of maize chitinases and the putative maize chito-oligosaccharide (PRR) receptor (*Zm*CEBiP), and the fungal effectors *Fv*cmp, *Fv*sep, and *Fv*CFEM. We selected these fungal effectors (*Fv*cmp, *Fv*sep) since they have a role in degrading plant chitinases, or in suppression of programmed plant cell death during pathogen response (*Fv*CFEM). We hypothesized that the bacterial control agent B25 may contribute to the regulation of maize chitinase gene expression during *Fv* infection, while also influencing the expression of plant cell wall-related and fungal effector genes.

## 2. Materials and Methods

### 2.1. Bacterial and Fungal Growth

The bacterial strain *Bacillus cereus sensu lato* B25 was previously isolated from the maize rhizosphere and maintained at −80 °C in Luria–Bertani (LB) medium as a 15% glycerol stock [10]. This bacterial strain has been reported to be non-hemolytic, suggesting it is not pathogenic to humans [10]. The bacterium was re-animated in LB agar at 28 °C for 16 h. *Fusarium verticillioides* strain P03 [6] was grown in potato dextrose agar (PDA) at 24 °C for fourteen days to induce conidia production. Conidia were collected using sterilized water and a Drigalski loop and adjusted to 1 × 10^6^ conidia/mL. B25 was grown in 5 mL of LB liquid medium for 16 h at 28 °C. A 250 mL baffled flask containing 100 mL of LB liquid medium was inoculated with 1 mL of B25 pre-inoculum (1 × 10^8^ UFC/mL) and grown at 28 °C for 24 h. B25 spore powder formulation was obtained [25].

### 2.2. B25 and Fv Colonization Assay in Maize Roots

To visualize the endophytic location of B25 and *Fv* inside the maize roots, a bipartite interaction assay was performed for each of the plant–microorganism interactions. A total of 200 grams of sterile sand was used as substrate containing 1 × 10^5^ *Fv* conidia per gram for the maize/*Fv* interaction. White maize seeds (Asgrow hybrid “Hipopótamo”) had been previously disinfected [6]. The maize seeds were treated with 0.2 mL of 1% (*w*/*v*) carboxy methyl cellulose (CMC) solution used as an adhesive agent. Seeds were coated with 1 g of B25 powder formulation spores (1 × 10^9^ spores g^−1^), obtaining a coating of ~1 × 10^6^ UFC/seed [25]. Control maize plants and maize/*Fv* plants were inoculated with the base powder formulation without B25. The experiment consisted of three biological replicates, each one containing three inoculated seeds per magenta box. The boxes were incubated at 25 °C for 16 h of light and 8 h of darkness at 20 °C. After two weeks, the plants were harvested, and roots were taken for further analysis. For confocal laser microscopy, the entire root system of three B25- or *Fv*-inoculated plants was inspected to confirm either bacterial colonization or fungal infection in several roots; after that, five representative roots of each plant were selected for capturing the best images. The wheat germ agglutinin (WGA) conjugated to the fluorophore Alexa Fluor 488™ was used to stain the cell walls of both microorganisms. Root samples were red fluorescence counter-stained with rhodamine B (plant cell wall staining). A Leica TCS SP5 confocal laser microscope with a white laser was used for capturing the green fluorescence emitted at 511–541 nm by WGA-488 after excitation at 499 nm, and the bacterial colonization counterstain was performed with rhodamine B by exciting it at 573 nm and registering emission at 593–623 nm. In the analysis of fungal infection, the counterstain was performed with propidium iodide excited at 499 nm and the red fluorescence emitted at 500–540 nm was captured. In addition, 100 mg of fresh root tissue was superficially disinfected to quantify endophytic B25 colony-forming units (CFU). Roots were washed with tap water and surface-sterilized by sequential immersion in 70% ethanol for 1 min and 1% hypochlorite for 10 min. Samples were then immersed in 10% (*v*/*v*) Tween-20 solution for 1 min and rinsed three times with sterile distilled water. The last wash was used directly on a plate as a control to demonstrate the efficiency of the surface sterilization procedure. Subsequently, root tissues were ground in a mortar containing 1 mL of sterile distilled water. Serial dilutions were prepared and plated on LB agar for bacterial quantification.

### 2.3. Rolled Paper Assay for Tripartite Interaction

An independent experiment was carried out using surface-disinfested white maize seeds (Asgrow hybrid “Hipopótamo”) coated with the B25 powder formulation as described in the previous section. For the rolled paper assay [26], four treatments were established for each time-course point: (1) control maize seeds (inoculated with base powder formulation without B25 spores); (2) maize seeds inoculated with a *B. cereus* B25 spore powder formulation; (3) maize seeds inoculated with 10 μL of *Fv* conidia suspension (1 × 10^3^ conidia/seed) and base powder formulation without B25 spores; (4) maize seeds inoculated with both microorganisms. The treatments were maintained in a growth chamber with controlled conditions at 25 ± 2 °C with a light–dark cycle of 14 h of light and 10 h of darkness. The maize seeds were watered daily with 10 mL of sterile distilled water per rolled paper. Maize roots were collected at 5, 7, 10, and 14 days post-inoculation (dpi). Each treatment had two biological replicates with four plants per replicate for every time point measured. A disease rating system [24] was employed in which 0 = 0% root rot; 1 = 1–25% root rot; 2 = 26–50% root rot; 3 = 51–75% root rot; 4 = 76–100% root rot; 5 = root system death; and 6 = whole seeding death. Length and fresh weight measurements of plant roots and shoots were also taken. Roots were ground in liquid nitrogen for RNA isolation.

### 2.4. Primer Design for Chitinase Detection

The design of the primers for the real-time PCR assays was carried out using the PRIMER3Plus software website [27]. The primers were designed using the coding sequence (CDS) of the genes and the 3′UTR region to obtain target gene specificity for each chitinase gene [23]. The housekeeping genes of maize, the cyclin-dependent kinase (*Zm00001eb350890*) and *Fv* β-tubulin, were used as internal control expression genes (Table 1).

### 2.5. RNA Extraction and cDNA Synthesis

Total RNA was isolated from roots of four plants from each biological replicate at each time point. The root samples were ground in liquid nitrogen using a mortar and pestle until a fine powder was obtained. TRIzol was used to obtain total RNA from all root samples from 100 mg of tissue for maize gene expression analysis. For *Fv* gene expression analysis, total RNA was obtained from root samples with *Fv* infection using the RNeasy Plant Mini Kit (Cat. No. 74904, QIAGEN). RNA was quantitated using a Nanodrop 2000c (Thermo Fisher Scientific, Wilmington, DE, USA) and RNA integrity determined by agarose gel electrophoresis. A total of 1 μg of RNA was treated with 1 unit of RQ1 DNase (PROMEGA, Cat. No. M6101, Fitchburg, WI, USA) to avoid DNA contamination. First-strand cDNA was synthesized from total RNA using oligo(dT) and SuperScript III reverse transcriptase (Thermo Fisher Scientific, Cat. No. 18080-044, Waltham, MA, USA) following the manufacturer’s instructions. Finally, cDNA was adjusted to a concentration of 10 ng/μL and a 1 μL aliquot was used for real-time PCR (qPCR) analyses.

### 2.6. Quantitative Real Time-PCR (qRT-PCR)

qRT-PCR reactions were performed using QuantiNova™ SYBR PCR kit (Qiagen, Cat. No. 208052, Hilden, Germany) in a reaction that included 5 μL of SYBR Green, 0.2 mM of each primer, 10 ng of cDNA and RNase-free water for a final volume of 10 μL. Negative controls were set up without any cDNA template, and no amplification was obtained in those. Each reaction was carried out in triplicate for each biological replicate. The reaction was carried out in a Rotor Gene-Q Real Time PCR System (Qiagen, Cat. No. 9001550, Hilden, Germany) with a thermocycler program included a preheating step at 95 °C for 2 min, followed by 40 cycles of denaturation at 95 °C for 30 s, an annealing step at 60 °C for 30 s, and an extension step at 72 °C for 30 s. Relative quantification of each gene was normalized using the maize cyclin-dependent kinase (CKD) gene, and the comparative threshold cycle method 2^−∆∆Ct^ [28] was used to calculate the fold change (FC) values. For *Fv* genes, the relative quantification was normalized using the *Fv* β-tubulin gene, and the comparative threshold cycle method 2^−∆Ct^ was used, and for the comparative analyses of maize–Fv vs maize–B25–Fv treatments, the threshold cycle method 2^−∆∆Ct^ was used. Melting curves indicated primer pairs were adequate for qRT-PCR analysis since they did not show any double amplifications (Figure S4). A standard curve was generated using serial 10-fold dilutions of cDNA for maize genes and genomic DNA for *Fv* genes. Amplification efficiency (E) and regression coefficient (R^2^) were calculated from the slope of the standard curve (Figure S5). All interaction graphics were designed using Biorender (https://www.biorender.com).

### 2.7. Protein–Protein Interaction Network

The STRING database (http://string-db.org) was used to generate a co-expression network [29]. The Zm00001eb317090 protein sequence was submitted to the STRING database. The minimum required score was set to medium confidence (0.400). The maximum number of interactors showed no more than 20 on the first shell, and no more than 10 on the second shell.

### 2.8. Statistical Analysis

Rolled paper assays were analyzed by one-way ANOVA using the IBM SPSS Statistics v. 25.0 program. Differences among treatments were determined using the Tukey test at a significance level of *p* < 0.05. The disease severity index data was arcsine-transformed before being analyzed using Student’s *t*-test (*p* ≤ 0.05) for comparison between the two treatments for each evaluated day.

## 3. Results

### 3.1. Biocontrol of Fv in Maize Plants by B25 Powder Formulation Spores

**Figure 1:**
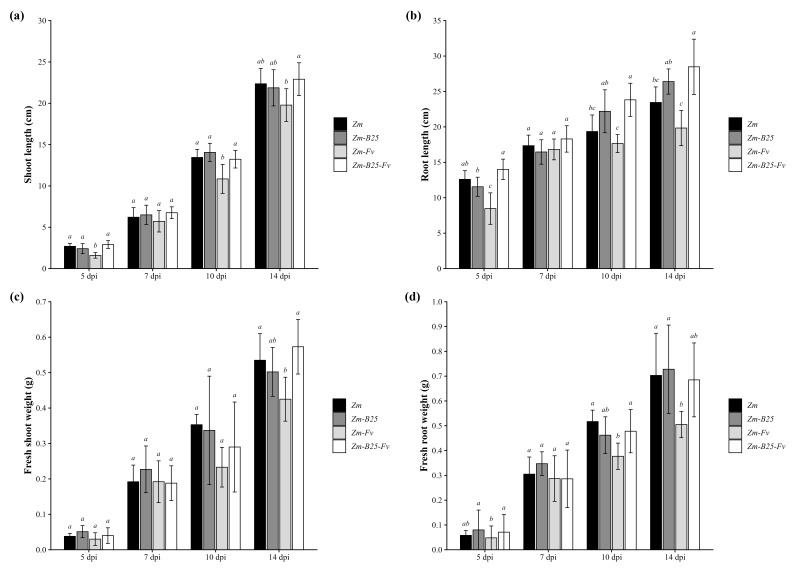
Growth of maize plants (*Zm*) in bi- and tripartite interactions with B25 and *Fv* in a time-course experiment. (**a**) Shoot length; (**b**) root length; (**c**) fresh shoot weight; (**d**) fresh root weight. Different letters indicate significant differences (*p* < 0.05) according to Tukey’s test. Values indicate the average of eight plants per treatment. Bars represent the mean values ± SE.

**Figure 2:**
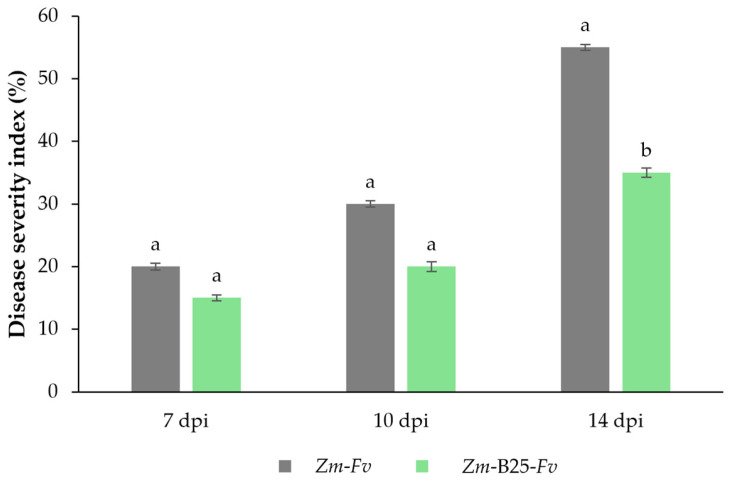
Effect of *Bacillus cereus* B25 on the disease severity of maize–*Fv* infection. Bars represent the disease severity index as a percentage (%). Different letters indicate significant differences (*p* ≤ 0.05) according to Student’s *t*-test. The statistical analyses were performed using the values for each day separately. Data are presented as mean ± standard deviation.

B25 controlled *Fv* infection in maize plants in different treatments in the time-course infection days (Figure 1). *Fv* infection reduced shoot length at 5 and 10 dpi compared to the other treatments and reduced shoot fresh weight at 14 dpi compared to *Zm* and *Zm*-B25-*Fv* treatment. *Fv* also caused a significant reduction in root length at 5 dpi, and in root fresh weight at 5, 10, and 14 dpi compared to the *Zm* control. In maize plants inoculated with B25 and *Fv* (*Zm*-B25-*Fv*), there was a significant difference in root length compared to the *Zm*-*Fv* treatment at 5, 10, and 14 dpi, and the control (*Zm*) treatment at 10 and 14 dpi. Likewise, no significant differences were detected at 7 dpi. Maize seeds treated with *Fv* only showed a disease index starting at 7 dpi. At 14 dpi, the disease severity index was significantly higher in the *Zm*-*Fv* than in the *Zm*-B25-*Fv* treatment (Figure 2).

### 3.2. B25 Endophytically Colonizes the Maize Root

**Figure 3:**
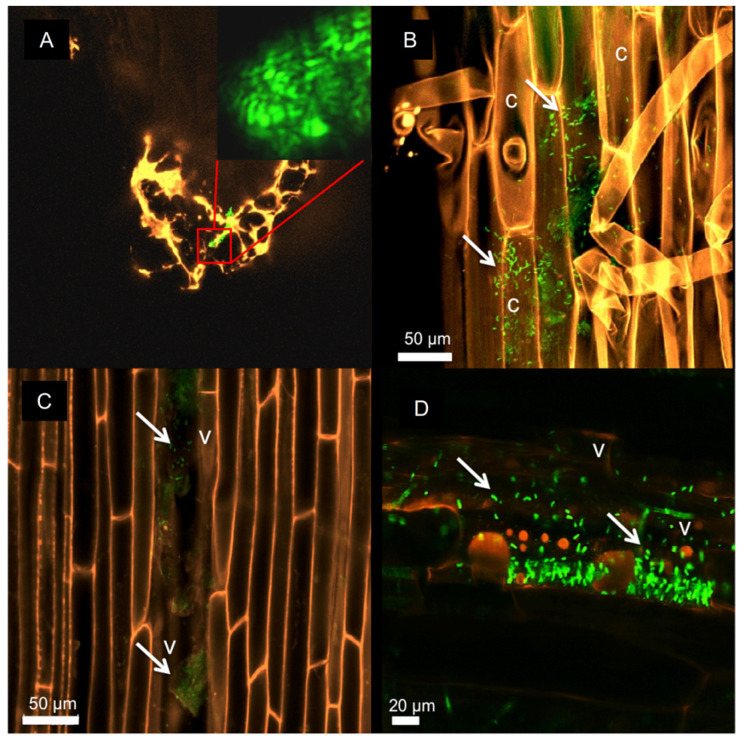
Confocal laser scanning microscopy showing the endophytic location of B25 and *Fv* inside vascular vessels at 14 dpi. (**A**) *Bacillus cereus* B25 colonization in the root apex. (**B**) *Bacillus cereus* B25 colonization in the root cortex. (**C**) *Bacillus cereus* B25 colonization in the root vessels. (**D**) *Fusarium verticillioides* colonization in the root vessels. Arrows indicate the bacteria cells (**A**–**C**), and fungal structures (**D**). Letter “c” indicates cortex cells and “v” indicates vascular cells.

B25 formed biofilm in the root apex (Figure 3A) and was present in the root epidermis (Figure 3B) and in the root vessels (Figure 3C). We observed that both B25 and *Fv* (Figure 3D) grew endophytically in the root vessels, and *Fv* and B25 independently colonized plants at 14 dpi, sharing the same niche inside the plant. We were able to observe B25 moving actively in the xylem vessels (Video S1). Likewise, endophytic bacterial abundance after two weeks reached 4.7 × 10^4^ CFU/100 mg of disinfected fresh root, while last-wash controls showed no bacterial growth, confirming the efficacy of the surface sterilization procedure and the endophytic nature of the bacterium.

**Figure 4:**
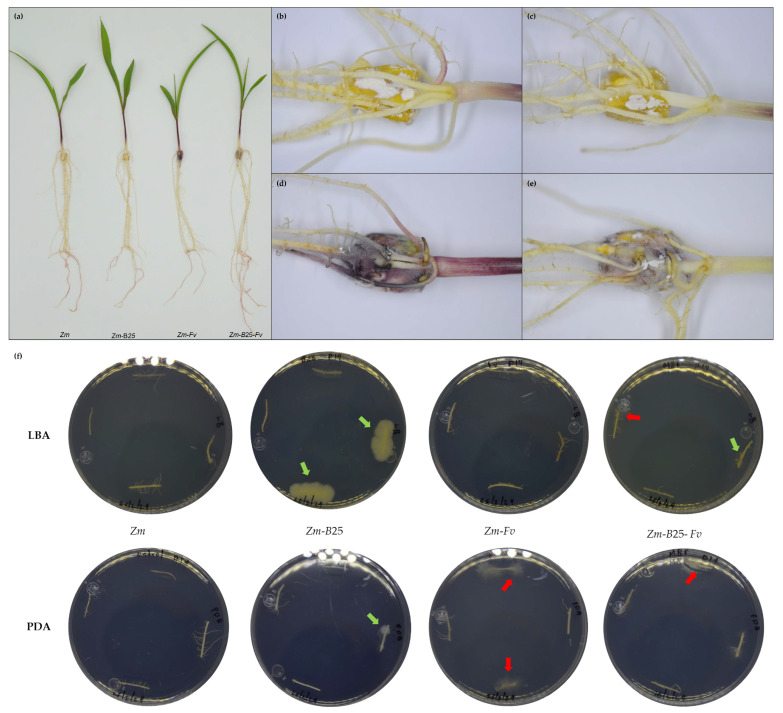
Growth of maize seedlings and detection of endophytic microorganisms at 14 dpi. (**a**) Maize seedlings from each treatment; (**b**) close-up of maize control seed; (**c**–**e**) close-up of inoculated maize seed with B25 (**c**), *Fv* (**d**) and both microorganisms (**e**); (**f**) growth of microorganisms from superficially disinfected root samples. Green arrows indicate B25 growth, and red arrows indicate *Fv* growth. LBA: Luria–Bertani Agar. PDA: Potato Dextrose Agar.

We also tested the endophytic presence of the microorganisms inside root samples by placing surface-sterilized root segments in LB and PDA plates, finding typical bacterial or fungal growth (Figure 4and Figures S1–S3). B25 and *Fv* were recovered from root samples at all sampling times. More abundant B25 colonies were observed in the *Zm*-B25 interaction, whereas reduced *Fv* mycelia growth was observed in the tripartite (*Zm*-B25-*Fv*) compared to the *Zm*-*Fv* bipartite interaction.

### 3.3. Differential Gene Expression and Protein–Protein Interaction Analysis

All chitinase genes were upregulated at some *Fv* infection time point (7, 10 and 14 dpi) (Table 2and Table S1) in the bi- and tripartite interactions. However, the highest upregulated gene was *bk4*—brittle stalk (*Zm00001eb317090*)—followed by *CEBiP* (*Zm00001eb002690*) and chitinase-29 (*Zm00001eb168350*). *bk4—*brittle stalk—was highly expressed in *Zm*-B25 and *Zm*-B25-*Fv* compared to its expression in *Zm*-*Fv*. Chitinase-29, a cytoplasmic chitinase of unknown function, was induced at 5 dpi in all interactions, at 7 dpi it was only induced in the *Zm*-B25 and *Zm*-B25-*Fv* interactions, at 10 dpi this gene was upregulated in the *Zm*-*Fv* treatment, and at 14 dpi it showed induction only in the tripartite interaction. Chitinase-27 (*Zm00001eb167340*), an extracellular chitinase which does not contain the protein domain recognized by the chitinase-modifying proteins, was induced at 5 dpi in the bipartite treatments, at 7 dpi in the *Zm*-B25 and *Zm*-B25-*Fv* interactions, at 10 dpi in the bipartite interactions, and in all conditions at 14 dpi with the highest induction in the *Zm*-B25 treatment.

Four fungalysin (*Fv*cmp)-targeted maize chitinases were analyzed: (1) ChitA (*Zm00001eb078730*) was upregulated at 10 dpi, being mostly induced by *Fv*, and at 14 dpi, the *Zm*-*Fv* treatment was the only one induced. (2) ChitB (*Zm00001eb425600*) was induced by day 7 in the tripartite treatment, at 10 dpi in the *Zm*-B25 treatment, and at 14 dpi in all treatments. (3) Chitinase-21–class I (*Zm00001eb346860*), which also has an *Fv*cmp site, was highly expressed at 7 dpi and remained induced at 10 dpi in the *Zm*-*Fv* and *Zm*-B25-*Fv* interactions, and at 14 dpi it was slightly induced only in the *Zm*-B25 interaction. (4) EPR4 (*Zm00001eb246640*) was induced at 7 and 14 dpi in all interactions and induced only in the bipartite interactions at 10 dpi.

Class I chitinases are proposed to be targeted not only by fungalysin but also by subtilisin-like proteins [30]. Therefore, a search was conducted to find a gene ortholog of *F. oxysporum* (*FOX_09801*) in the *Fv* genome. An orthologous gene was found with 95.98% nucleic acid identity *(FVEG_08679*) to the one from *F. oxysporum* and it was denominated *Fvsep* (serine protease). *Fvsep* (*FVEG_08679*) was upregulated at 7 dpi in *Zm*-B25-*Fv* with a fold change of 2.50. The *Fv CFEM* gene, coding for a putative effector possibly involved in suppressing chitin-triggered reactive oxygen species (ROS) accumulation, was induced in all interaction days in *Zm*-*Fv* with a high FC value at 14 dpi. However, this gene was also induced in the tripartite interactions at 5, 10 and 14 dpi. *Fvcmp* always showed low levels of expression (Table S2).

In addition, the putative maize chito-oligosaccharide (PRR) receptor *CEBiP* was induced from 5 dpi onwards but the induction was highest in *Zm*-B25 and *Zm*-B25-*Fv* compared to *Zm*-*Fv* treatment and continued at 7 dpi in the *Zm*-B25 and *Zm*-B25-*Fv* treatments and at 10 dpi only in the *Zm*-B25 interaction.

**Figure 5:**
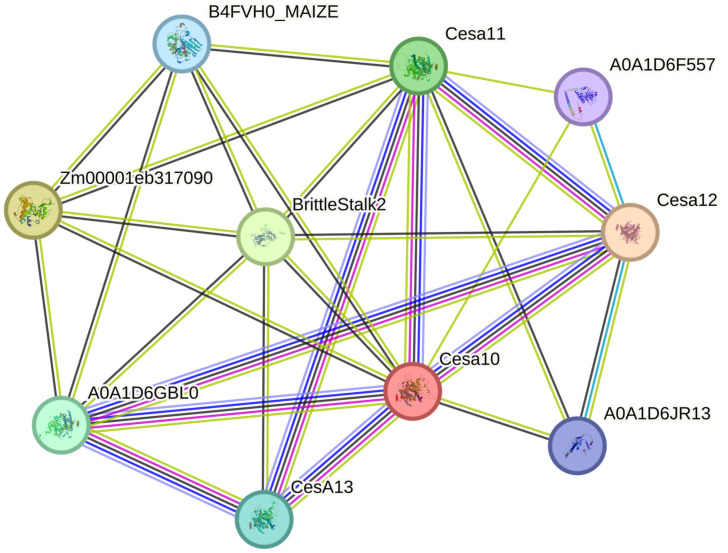
Predicted protein interaction network between Zm00001eb317090 and other maize proteins by STRING analysis. Blue lines: protein homology; violet lines: experimentally determined; navy blue lines: gene co-occurrence; yellow lines: textmining; black lines: co-expression; aqua blue: from curated databases. Colored nodes represent each protein’s interactors.

A protein interaction analysis was performed to predict which genes interact with brittle stalk (*Zm00001eb317090*) due to the high induction of this gene (Figure 5). We found nine possible protein interactors: bk2 brittle stalk, B4FVH0, A0A1D6JR13, A0A1D6F557 and five cellulose-synthase (CesA10, 11, 12, 13 and 14—A0A1D6GBL0) proteins. Most of these proteins may be involved in cell wall biosynthesis. B4FVH0 (*Zm00001eb397000*) is proposed to encode an O-acetyltransferase involved in cell wall formation; A0A1D6JR13 (*Zm00001eb138390*) and A0A1D6F557 (*Zm00001eb112280*) are plant-specific domain TIGR01627 family protein members involved in xylan biosynthesis.

STRING analysis predicted *bk2 brittle stalk* as a gene hub involved in cell wall synthesis/strengthening (Figure 5). Thus, we analyzed its expression by qRT-PCR and found out that this gene is upregulated in the *Zm*-B25 associations at 7 dpi compared to the other treatments, with *Zm*-*Fv* being the one with the lowest gene expression (Table 3, Table S1).

## 4. Discussion

*Fusarium verticillioides* is considered an endemic fungus of maize fields and it can prevail in soils as conidia or spores for many years [4]. In addition, *Fv* can infect other important agricultural crops: not only maize, but also tomato [31], potato [32], sorghum [33], and sugarcane [34]. Over the years, great advances have been made regarding the mechanisms of tolerance of maize to *Fv* infection through cultivation techniques of tolerant genotypes, which have allowed us to elucidate the molecular mechanisms underlying the maize–*Fv* interaction and how *Fv* can infect maize tissues. For example, the *ZmWAX* gene is involved in maize resistance to *Fv* seed and stalk rot and cuticular wax deposition [35].

A control agent for SERR caused by *Fv*, a bacterium from the maize rhizosphere, *Bacillus cereus* B25, was identified and demonstrated *in vitro* to control *Fv* disease [10,11]. B25 also controlled SERR symptoms caused by *Fv* in field trials where it showed the ability to reduce fumonisins and increase grain yield [11]. The B25 genome was sequenced [12] and some of B25’s antagonistic mechanisms against *Fv* were suggested by identifying genes involved in the production of siderophores (bacillibactin and petrobactin), antibiotics (surfactin), lytic enzymes (endoglucanase and chitinases), and biofilm formation which were later corroborated by gene expression, physiological and biochemical studies [14]. B25 chitinases have been found to be induced in the presence of fungal lysates [13]; in addition, recombinant B25 chitinases were shown to have a negative effect on *Fv* conidia germination [15]. Therefore, to understand what the interaction mechanisms of B25 within the maize root cells are, and in the presence of *Fv*, we investigated if this bacterium could induce maize chitinase genes.

In the bipartite and tripartite assays, we observed the effect of B25 and *Fv* on maize seedling growth; the greatest growth in root length was obtained when *Fv* was combined with B25. Maize chitinases can participate in several plant biological processes such as seed germination, growth and fungal control [36]. The increase in plant chitinase gene expression has been observed in the presence of several phytopathogens, for example, the BjChI1 chitinase of *Brassica juncea* in response to *Aspergillus niger* [37], the chitinase (CHI) from *Vitis vinifera* L. in response to *Aspergillus carbonarius* [38] and the induction of chitinases in soybean by adding oligochitosan complexes in response to *Colletotrichum truncatum* [39]. The expression of chitinases in plants can be influenced by some symbiotic organisms, such as *Medicago truncatula* in the presence of *Glomus intraradices* [40] and soybean chitinases induced in response to different rhizobacteria inoculation [41]; this could lead to early detection and rapid response against some pathogens.

Regarding maize chitinase genes, *bk4 brittle stalk* presented the highest fold change value in all treatments in our study, especially in the bacterial treatments. Notably, this gene has been reported to be involved in stalk tensile strength and could be involved in cell wall regulatory formation [42]. Likewise, this gene is also related at the co-expression level with *bk2 brittle stalk*, which encodes for a COBRA-LIKE4 protein; this protein can function in a lignin–cellulosic interaction [43]. Therefore, both genes could be participating during the biosynthesis of cell wall compounds.

**Figure 6:**
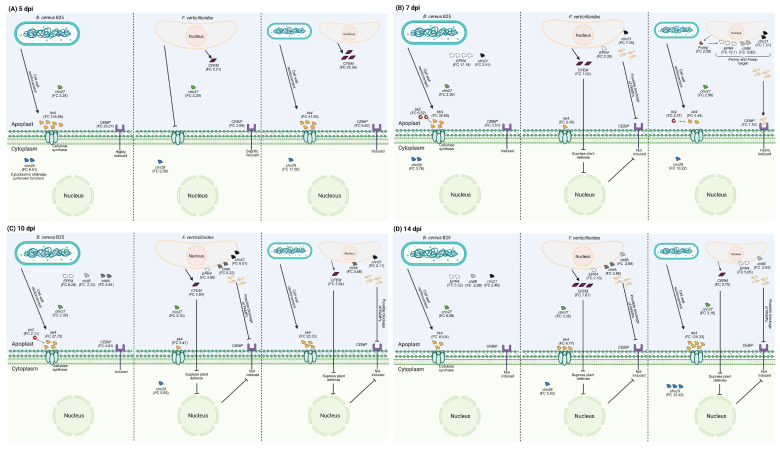
Proposed interaction model between maize, *Fusarium verticillioides* and *Bacillus cereus* B25 in bipartite and tripartite associations. B25 consistently induced chitinase-related cell wall genes (*bk2* and *bk4*) (**B**,**C**), both functionally associated with cellulose synthases, alongside *CEBiP*-mediated PAMP detection (**A**–**C**). *Fv* caused a decrease in *CEBiP* induction (**A**–**D**), likely through CFEM protein activity, while impairing cell wall reinforcement responses. In the tripartite interaction, B25 partially rescued *bk4* and *CEBiP* gene induction at early time points (**A**,**B**). Chitinase expressions (*chn27*, *chn29*, *EPR4* and *chitb*) varied depending on the time point and treatment. The fungal gene *fvcmp*, which encodes a chitinase-modifying protein, was induced when its target chitinases (*chitA*/*chitB*) were present, whereas *FvCFEM* was consistently upregulated in most *Fv* treatments.

Chitinase-27 (*Zm00001eb167340*) has been found to be induced in maize silk samples infected with *Fv* [44]. Chitinase-29 (*Zm00001eb168350*) is downregulated in leaf samples in susceptible maize plants infected with *Fv* [45]; interestingly, in this study, chitinase-29 was induced mostly in the bacterial treatments, suggesting that B25 may be regulating its expression, and since its induction was higher in the tripartite interaction, it may be being induced in response to *Fv* infection while interacting with B25. In addition, chitinase-21 (*Zm00001eb346860*), one of the *Fvcmp* target chitinases, has been found to be induced in the maize line (RIL165) which is susceptible to *Cercospora zeina* [46]. Combining this all together, we proposed an interaction model for chitinase-related gene expression between maize, B25 and *Fv* for each day of interaction (Figure 6).

In the presence of B25 bacteria, the highest induction of *CEBiP* receptor and the *bk4* gene at 5 dpi suggests the plant prepares to repel pathogen attack by plant cell wall reinforcement and preparedness for PAMP signal detection. The *chn27* gene, an extracellular chitinase, is induced at that time, which could degrade chitin from fungal pathogens in case they try to colonize the plant. The same scenario persists at days 7 and 10 with *bk2* being induced by B25 which could assist plant cell reinforcement even more. *CEBiP* remains induced and chn27 and different cmp-containing chitinases are induced at those times which should allow for PAMP generation in case of fungal invasion. Extracellular chitinases remain induced at day 14, as well as bk4. Only *CEBiP* is not induced but PAMP detection remains possible due to remaining *CEBiP* protein-sensing chito-oligosaccharides. In summary, B25 allows plants to get ready to fight fungal pathogens through plant cell wall reinforcement, PAMP generation and PAMP signal detection. It is plausible to suggest that B25 may be causing gene expression changes early on during colonization to avoid fungal invasion, which results in a lower severity index at day 14 in the *Zm*-*Fv*-B25 interaction (Figure 2).

*Zm-Fv* interaction by 5 dpi slightly induces *CEBiP*, *chn27* and *chn29*. This raises the following question: is the extracellular chitinase *chn27* able to degrade chitin from the fungal cell wall and induce PAMP detection by the plant? At this time, *bk4* is also not induced with the lack of reinforcement of the plant cell wall. The plant prepares to respond to *Fv* by inducing cmp-containing chitinases by 7 dpi; however, since *CEBiP* induction is also lost currently, it may be possible that *Fv* can affect PAMP detection by an unknow mechanism, possibly mediated by CFEM proteins. To respond to fungal attack, the plant slightly induces *bk4* expression, but this is possibly not enough to cause reinforcement of the plant cell wall. *Fv* could partially prevent gene induction of *bk2* at lower levels than the ones induced in the B25 treatments and prevent plant cell wall biosynthesis and reinforcement. Root biomass is affected by *Fv* from 10 dpi onwards; at this time point, the plant attempts to defend itself from fungal invasion, increasing the expression of three extracellular cmp-containing site chitinases, *chn27*, and the cytosolic chitinase *chn29*, suggesting the plant tries to rescue its mechanism of plant defense mediated by plant chitinases in order to fight against *Fv*. A similar gene expression scenario is encountered at 14 dpi where *Fv* also causes a noticeable decrease in root biomass and shoot biomass. In summary, maize can response to *Fv* infection by inducing several chitinase genes and cell wall-related chitinases; however, this response is not enough to prevent fungal colonization.

In the *Zm*-B25-*Fv* interaction at 5 dpi, only *bk4*, *CEBiP* and the cytosolic *chn29* are induced. These genes remained induced at 7 dpi, but at this time three cmp-containing site chitinases and *chn27* are also induced, possibly causing PAMP generation and detection; *bk2* is also induced enhancing plant cell wall reinforcement. At this point the fungus tries to protect itself from maize chitinases by inducing *fvsep*. As in the *Zm*-*Fv* interaction at 10 dpi, *CEBiP* is not induced, but in the *Zm*-B25-*Fv* interaction, *bk4* induction is higher and *chitA* and *chn21* remain induced. A different set of cmp-containing site chitinases were induced at 14 dpi: *chitB* and *EPR4*. *Chn27* was also induced, as well as *chn29* which was highly induced. A noticeable increase in gene expression at this time point was observed for *bk4* with respect to 10 dpi (120.33- vs. 25.33-fold change). The symptoms and disease severity index of the plants associated with both organisms are lower than in the maize associated with *Fv* only. It is interesting to note that the *Fv CFEM* gene was induced at 5 dpi with a high FC value in the *Zm*-B25-*Fv* interaction compared to the bipartite *Zm*-*Fv* interaction, suggesting that *Fv* tries counter-attacking B25’s antagonistic effect.

**Figure 7:**
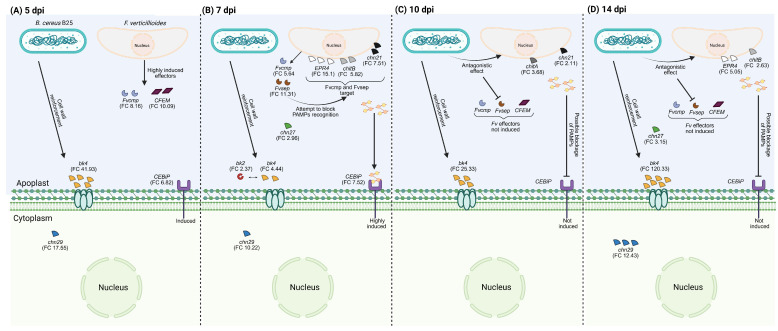
Proposed interaction model between maize–B25–Fv. (**A**) 5 dpi: B25 modulated early fungal responses by inducing *CFEM* and *fvcmp* genes, suggesting an initial adjustment of the fungal virulence mechanism. (**B**) 7 dpi: the interaction reaches a peak confrontation stage, with fungal expression shifting toward a proteolytic response, evidenced by high induction of *fvsep* and *fvcmp* genes, while *CFEM* is not induced. This may be related to the increase in maize chitinase-induced genes, suggesting that B25 enhances pathogen perception by increasing PAMP recognition. (**C**) 10 dpi: fungal gene effectors are not induced while maize chitinases and *bk4* induction are sustained, suggesting direct antagonistic activity by B25; notably, *CEBiP* remains uninduced despite the absence of CFEM, indicating a putative CFEM-independent immune suppression mechanism. (**D**) 14 dpi: fungal genes remain repressed alongside induction of chitinase cell wall-related gene *bk4*. This possibly suggests that B25 progressively limits *Fv* growth while promoting chitinase induction.

In order to understand the effect of B25 on *Fv* gene expression, a 2^−∆∆Ct^ analysis was performed comparing the expression levels of *Zm*-B25-*Fv* against *Zm-Fv* (Figure 7, Table S3).

In summary, when the tripartite interaction occurs in maize roots, B25 seems to slow down the advancement of *Fv* in the first 7 dpi by plant cell wall reinforcement and PAMP detection by inducing *CEBiP*; at 10 and 14 dpi, plant cell wall reinforcement mediated by *bk4* may continue in the plant. It is plausible to suggest that B25 could be producing chitinases that could directly affect the fungal cell wall at 10 and 14 dpi, since *Fvcmp* and *Fvsep* were highly induced at 7 dpi followed by a lack of induction at days 10 and 14 post-inoculation. However, B25 chitinase gene expression has eluded detection in our maize root system. The *Fv*sep subtilisin-like protein may be acting together in the tripartite interaction with the fungalysin *Fv*cmp to modify class I and IV chitinases [30], counteracting the plant response of induction of the extracellular chitinases which could degrade the fungal cell wall. In addition, *Fv* CFEM proteins have been described as a dual-function effector that suppresses plant immunity while contributing to fungal cell wall integrity during early stages of infection [47]; this could be associated with the high-level expression of the *FvCFEM* gene at 5 dpi. The induction of two B25 extracellular chitinases in response to chitin or fungal lysates [13], along with evidence that bacterial chitinases can decrease conidia germination [15] and mycelial branching of *Fv* [14] and that B25 and *Fv* appear to share the same niche in the root vascular tissue (Figure 3), suggests that when in contact inside the plant root, direct antagonistic mechanisms of B25 against *Fv* may help to debilitate the fungal defenses. Even when the fungus can respond by mounting an avoidance mechanism of PAMP signaling, B25 can help the plant in a coordinated dual defense. While the plant reinforces its cell wall, B25 uses all its weaponry against the fungus, including lytic fungal cell wall enzymes such as chitinases, chitosanase and endoglucanase, biofilm production, antibiotics (surfactin) and siderophores (petrobactin and bacillibactin), to delay fungal infection.

Our hypothesis on the contribution of B25 to the regulation of maize chitinase, plant cell wall-related, and fungal effector genes was accepted since the presence of this bacterial control agent regulated their expression under the different bi- and tripartite interactions. The proposed mechanisms derived from this work provide valuable insights into the potential mechanisms involved in the tripartite maize–*Fv*–B25 interaction but were inferred only from gene expression analyses and protein prediction approaches, which represents a limitation of this study. Protein recombination studies are still needed to further elucidate the effects of *Fv* proteases, such as *Fv*sep, on maize chitinases. In addition, direct protein–protein interaction analyses would be necessary to assess the potential interaction between *bk4* and cellulose synthase proteins along with the possible effect of B25 on cell wall reinforcement.

## 5. Conclusions

In summary, maize chitinase genes were analyzed to learn about their expression patterns in a time-course interaction assay involving the biocontrol bacterium B25, the fungal phytopathogen *Fv* or both together. Plant physiological analyses suggest that B25 plays a major role in controlling *Fv* infection. Furthermore, we demonstrate that B25 can grow endophytically inside the vasculature of maize roots, just like *Fv*. Additionally, maize chitinases may regulate the MAP kinase cascade through the production of PAMPs and the activation or repression of the *ZmCEBiP* chito-oligosaccharide receptor. Meanwhile, during *Fv* infection, CFEM, *Fv*cmp and *Fv*sep are induced; these last two could act in a coordinated fashion to modify class I and IV extracellular maize chitinases. Further analyses are needed to confirm that *bk4* induction by B25 will result on cell wall strengthening. These findings highlight the maize chitinase gene expression variability during bi- and tripartite interactions in the presence of a beneficial bacterium for controlling *Fv* infection, as well as provide an insight into the molecular mechanisms that B25 utilizes, which will help to explain the use of this strain which has already been validated as a plant growth-promoting and biocontrol agent in field experiments [11].

## Figures and Tables

**Table 1:** Primers used in this study.

Gene ID	Gene Name	Forward Primer (5′—3′)	Reverse Primer (5′—3′)
**Maize chitinases**			
*Zm00001eb393070*	*bk2*	ACTTGGGTTTTCGTCAGAGG	TGCCAATCTTGTAGGAGACG
*Zm00001eb317090*	*bk4*	TAGTTGCCACTTCGCTTTCC	AAGATCTCGCGGTTGTTGAG
*Zm00001eb346860*	*chn21*	CTACAAGCGCTACTGCGATG	CACACACACGTTTTCACTGC
*Zm00001eb167340*	*chn27*	ACGCGTACATGTTCCAGAAG	AGATCATGAGGCCACCGTAG
*Zm00001eb168350*	*chn29*	AAACAATCAGGGGTCCATCC	AGCTAACGAAGGCGTTGATG
*Zm00001eb078730*	*chitA*	TCACCTCACACAACAAGCTG	TACTGGGTTCACAGCGAACTAC
*Zm00001eb425600*	*chitB*	CAGTATGGCTATGGCAAAGG	ACAGCGCAGAGGAGTGATAG
*Zm00001eb246640*	*EPR4*	ACAACCTCACCTGCTGAATG	GCAATCGCCATCTATCCATC
**COs receptor**			
*Zm00001eb002690*	*CEBiP*	TAGACTGCACTCCGGTGAAAG	GGTGTTGGTATAACCGCTGTAAG
***Fv*** **genes**			
*FVEG_13630*	*Fvcmp*	GCACCAGCCTTACCA CTAACC	GCATCACTGTTCCCGTGC
*FVEG_08679*	*Fvsep*	GGCAGAATCACTGGTACTCTC	TGAACCCTTCGCATTTACG
*FVEG_07535*	*CFEM*	ATGGCCCTTGCTCTGTAAAC	AACAATGCCTGTCACCTCAC
**Housekeeping genes**			
*Zm00001eb350890*	*cdk*	CCGTCATCGCCTCACGAAGAG	AGAGCCTGCCTTACGGAATTG G
*Fvtub*	*β-tubulin*	ACATCCAGACAGCCCTTTGTG	AGTTTCCGATGAAGGTCGAAGA

**Table 2:** Quantitative real-time PCR expression analysis of maize chitinases and the maize oligomer chitin-receptor, and fungalysin, subtilisin, and CFEM from *Fusarium verticillioides*.

	Genes										
Treatment	*Zm167340* chn 27	*Zm168350* chn 29	*Zm317090* bk4	*Zm346860* chn 21	*Zm078730* chitA	*Zm425600* chitB	*Zm246640* *EPR4*	*Zm002690* *CEBiP*	*FVEG_13630* *Fvcmp*	*FVEG_08679* *Fvsep*	*FVEG_07535* *CFEM*
	**5 dpi**										
***Zm*** **-** ***B*** **25**	3.24	6.01	145.68	1.14	0.77	0.21	0.16	23.21	-	-	-
***Zm-Fv***	2.29	2.59	0.52	0.86	0.67	0.01	0.5	2.09	0.12	1.35	2.21
***Zm*** **-** ***B*** **25-** ***Fv***	1.81	17.55	41.93	0.79	1.86	0.09	0.23	6.82	0.98	0.49	22.34
	**7 dpi**										
***Zm*** **-** ***B*** **25**	2.36	3.76	38.65	2.51	1.28	1.71	17.19	3.51	-	-	-
***Zm-Fv***	0.81	1.11	2.45	7.45	0.47	0.54	2.35	0.79	0.12	0.22	7.03
***Zm*** **-** ***B*** **25-** ***Fv***	2.96	10.22	4.44	7.51	0.50	5.82	15.1	7.52	0.67	2.50	1.54
	**10 dpi**										
***Zm*** **-** ***B*** **25**	2.39	0.37	27.73	1.05	5.81	2.2	6.28	4.81	-	-	-
***Zm-Fv***	2.10	5.63	3.47	8.51	8.22	1.32	3.96	0.8	1.02	0.65	3.54
***Zm*** **-** ***B*** **25-** ***Fv***	1.25	1.28	25.33	2.11	3.68	0.59	1.42	1.71	0.44	0.61	3.94
	**14 dpi**										
***Zm*** **-** ***B*** **25**	6.58	0.21	10.04	2.46	0.32	2.09	5.52	0.11	-	-	-
***Zm-Fv***	3.36	0.09	6.77	1.10	3.86	2.68	2.16	0.1	0.17	0.80	7.67
***Zm*** **-** ***B*** **25-** ***Fv***	3.15	12.43	120.33	0.86	0.26	2.63	5.05	0.37	0.12	0.66	3.75

The cyclin-dependent kinase gene was used for normalization of maize genes and β-tubulin for *Fv* genes. *Fv* genes were analyzed using the 2^−∆Ct^ method. (-) refers to undetectable expressions due to the lack of the presence of *Fv*.

**Table 3:** Quantitative real-time PCR expression analyses of *brittlestalk2* in all treatments.

Treatment	5 dpi	7 dpi	10 dpi	14 dpi
*Zm*-*B*25	1.42	6.52	2.11	0.83
*Zm-Fv*	0.53	0.85	1.47	0.35
*Zm*-*B*25-*Fv*	1.41	2.37	0.70	1.40

The cyclin-dependent kinase gene was used for gene normalization. The FC value was obtained from 2^−∆∆Ct^ analysis.

## Data Availability

The original contributions presented in this study are included in the article/Supplementary Material. Further inquiries can be directed to the corresponding author.

## Supplementary Materials

The following supporting information can be downloaded at https://www.mdpi.com/article/10.3390/microorganisms14071517/s1. Figure S1: Growth of maize seedlings and microorganisms at 5 dpi; Figure S2: Growth of maize seedlings and microorganisms at 7 dpi; Figure S3: Growth of maize seedlings and microorganisms at 10 dpi; Figure S4. Melting curves of fourteen primer pairs of genes analyzed by real-time PCR; Figure S5. Standard curves of fourteen genes analyzed by real-time PCR to calculate amplification efficiency; Table S1: Quantitative real-time PCR expression (2^−∆∆Ct^) of maize chitinase genes; Table S2: Quantitative real-time PCR expression (2^−∆Ct^) of *Fusarium verticillioides* genes; Table S3: Quantitative real-time PCR expression (2^−∆∆Ct^) of *Fusarium verticillioides* genes; Video S1: Endophytic growth of B25 inside xylem vessel cells.
