# Supplementary material for: Biological Control of Fusarium verticillioides P03 in Maize by Bacillus cereus sensu lato B25 Involves Coordinated Host–Bacterium Responses
Source: Microorganisms. 2026 Jul 11;14(7):1517. doi: 10.3390/microorganisms14071517 (PMC13413985; doi:10.3390/microorganisms14071517)
Supplement: Supplementary file 1 [file microorganisms-14-01517-s001.zip › Supplementary figures.pdf]

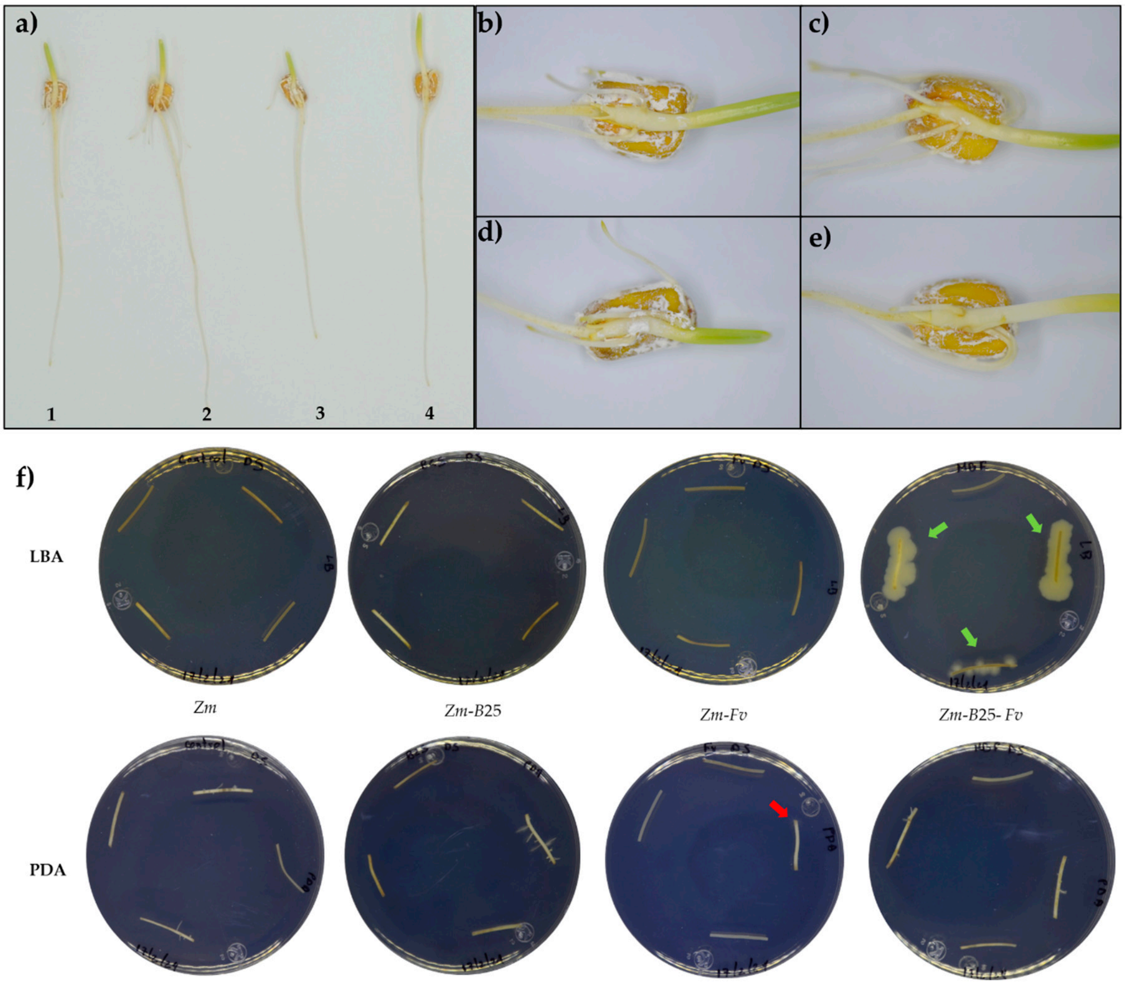

Supplementary Figure S1. Growth of maize seedlings and microorganisms at 5 dpi. A) maize seedlings of each treatment; b-c) close-up of inoculated seed from *Zm* (b) and *Zm-B*25 (c); d-f) close-up of inoculated seed from *Zm-Fv* (c) and *Zm-B*25-*Fv* (d); f) growth of microorganisms around root samples. Green arrows indicated *B*25 growth and red arrows *Fv* growth. LBA: Luria Bertani Agar. PDA: Potato Dextrose Agar.

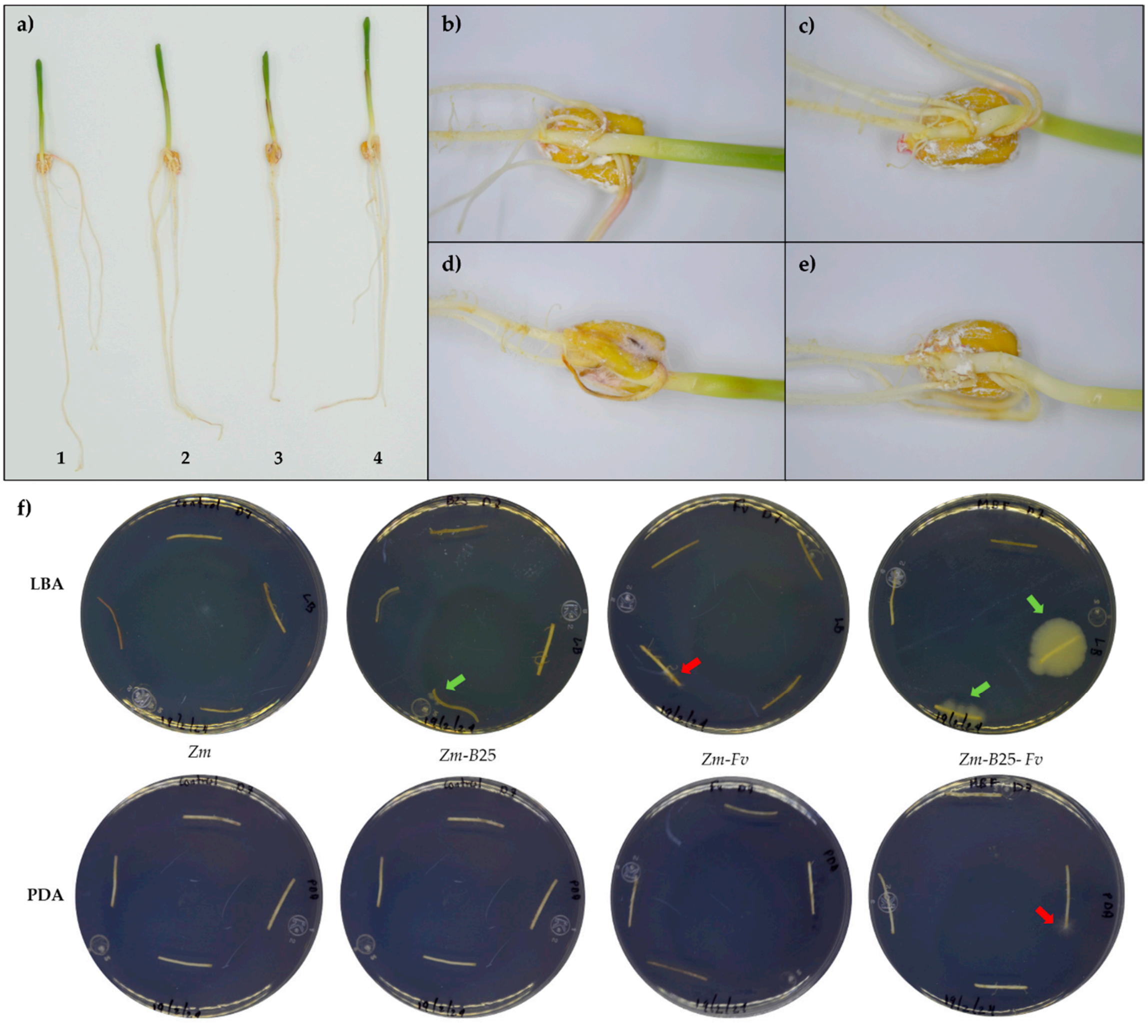

Supplementary Figure S2. Growth of maize seedlings and microorganisms at 7 dpi. A) maize seedlings of each treatment; b-c) close-up of inoculated seed from *Zm* (b) and *Zm-B25* (c); d-f) close-up of inoculated seed from *Zm-Fv* (c) and *Zm-B25-Fv* (d); f) growth of microorganisms around root samples. Green arrows indicated *B25* growth and red arrows *Fv* growth. LBA: Luria Bertani Agar. PDA: Potato Dextrose Agar.

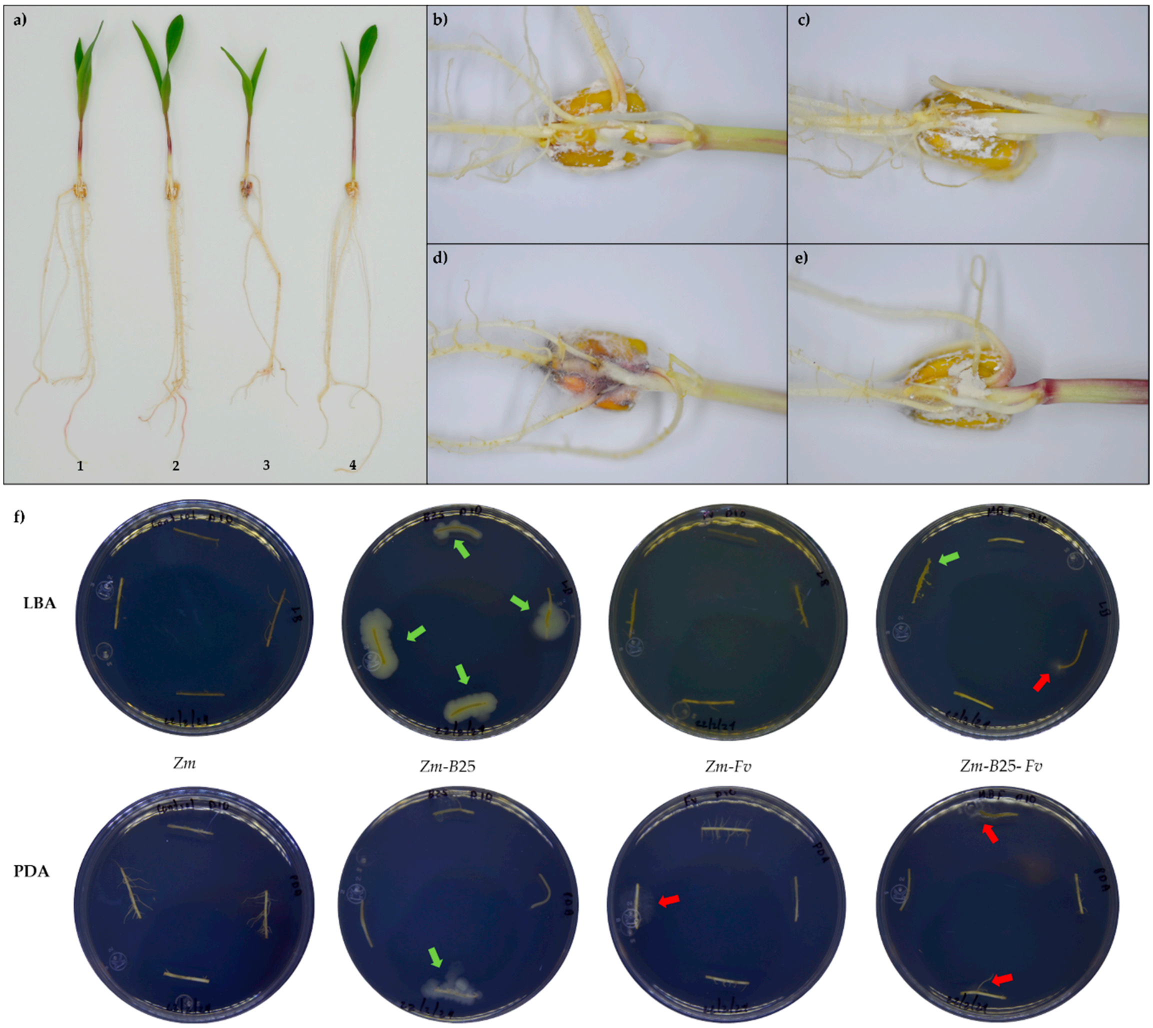

Supplementary Figure S3. Growth of maize seedlings and microorganisms at 10 dpi. A) maize seedlings of each treatment; b-c) close-up of inoculated seed from *Zm* (b) and *Zm-B*25 (c); d-f) close-up of inoculated seed from *Zm-Fv* (c) and *Zm-B*25-*Fv* (d); f) growth of microorganisms around root samples. Green arrows indicated *B*25 growth and red arrows *Fv* growth. LBA: Luria Bertani Agar. PDA: Potato Dextrose Agar.

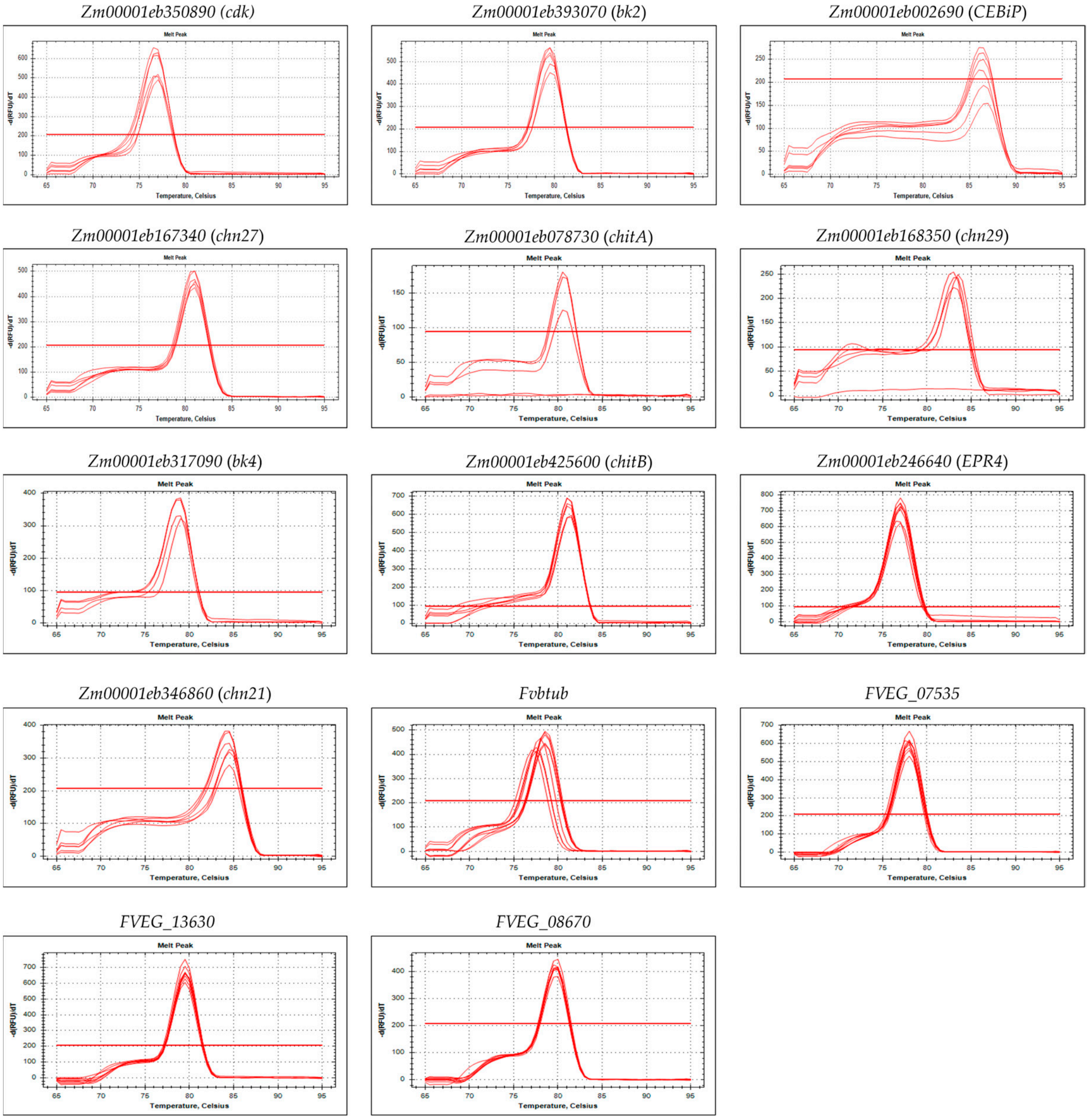

Supplementary Figure S4. Melting curves of the fourteen genes analyzed by quantitative real time PCR.

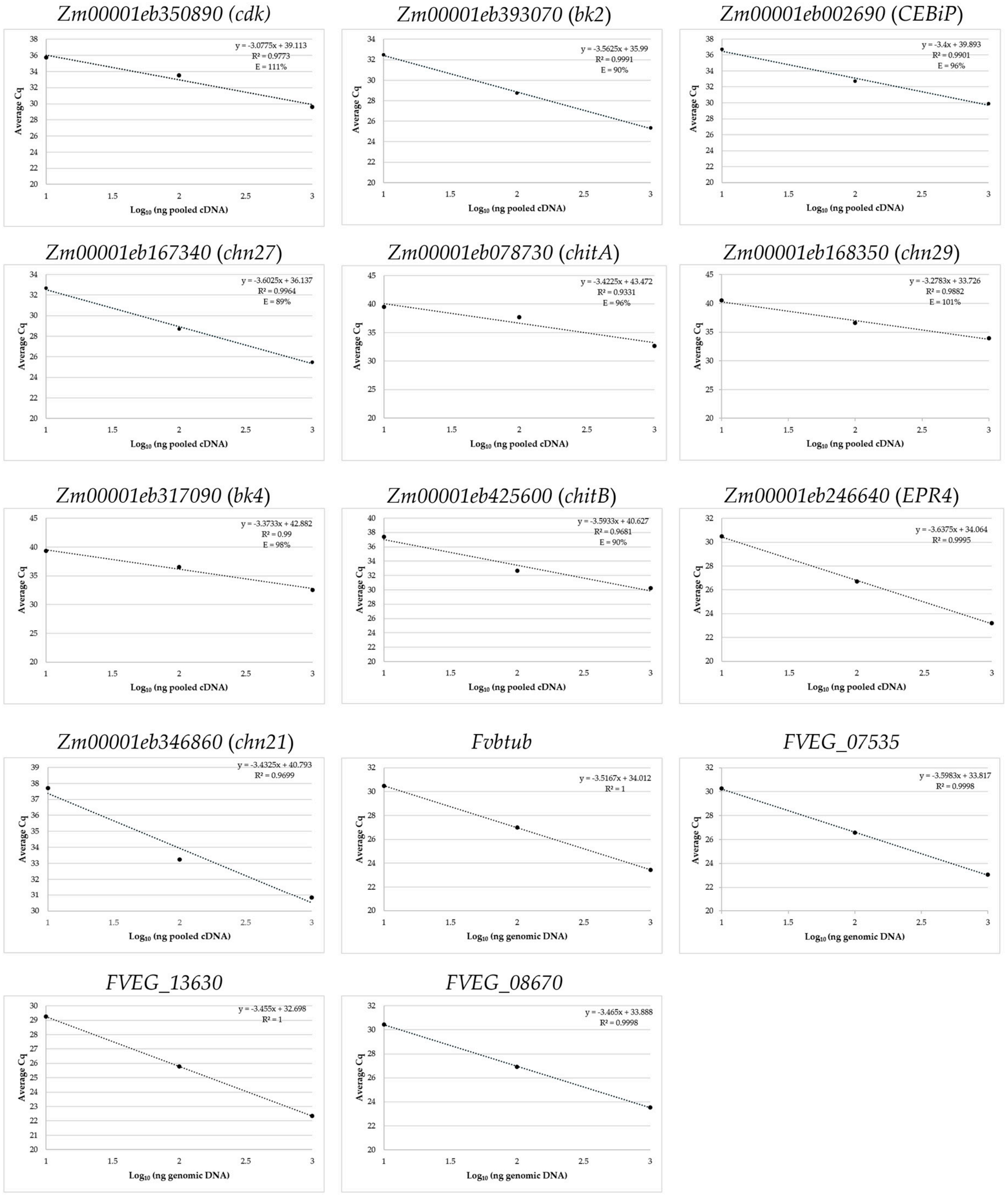

Supplementary Figure S5. Standard curve of the fourteen genes analyzed by real time PCR to calculate amplification efficiency.
